# Genetic Links between Endometriosis and Endometriosis-Associated Ovarian Cancer—A Narrative Review (Endometriosis-Associated Cancer)

**DOI:** 10.3390/life14060704

**Published:** 2024-05-30

**Authors:** Tanja Pejovic, Ann M. Cathcart, Rofieda Alwaqfi, Marjorie N. Brooks, Rachel Kelsall, Farr R. Nezhat

**Affiliations:** 1 Department of Obstetrics and Gynecology, Providence Medical Center and Providence Cancer Institute, Medford, OR 97504, USA; marjorie.brooks@providence.org; 2 Department of Obstetrics and Gynecology, Oregon Health & Science University, Portland, OR 97201, USA; cathcara@ohsu.edu; 3 Department of Pathology and Laboratory Medicine, Memorial Sloan-Kettering Cancer Center, New York, NY 10065, USA; alwaqfir@mskcc.org (R.A.); farr@farrnezhatmd.com (F.R.N.); 4 Pacific Northwest University of Health Sciences, Yakima, WA 98901, USA; rkelsall@pnwu.edu; 5 Weill Cornell Medical College, Cornell University, New York, NY 10065, USA; 6 NYU Long Island School of Medicine, Mineola, NY 11501, USA

**Keywords:** endometriosis, cancer, ARD1A, ovarian cancer, clear-cell carcinoma, endometrioid adenocarcinoma

## Abstract

Endometriosis is a frequent, estrogen-dependent, chronic disease, characterized by the presence of endometrial glands and stroma outside of the uterine cavity. Although it is not considered a precursor of cancer, endometriosis is associated with ovarian cancer. In this review, we summarized the evidence that clear-cell and endometrioid ovarian carcinomas (endometriosis-associated ovarian carcinoma—EAOC) may arise in endometriosis. The most frequent genomic alterations in these carcinomas are mutations in the AT-rich interaction domain containing protein 1A (ARID1A) gene, a subunit of the SWI/SNF chromatin remodeling complex, and alterations in phosphatidylinositol 3-kinase (PI3K) which frequently coexist. Recent studies have also suggested the simultaneous role of the *PTEN* tumor-suppressor gene in the early malignant transformation of endometriosis and the contribution of deficient MMR (mismatch repair) protein status in the pathogenesis of EAOC. In addition to activating and inactivating mutations in cancer driver genes, the complex pathogenesis of EAOC involves multiple other mechanisms such as the modulation of cancer driver genes via the transcriptional and post-translational (miRNA) modulation of cancer driver genes and the interplay with the inflammatory tissue microenvironment. This knowledge is being translated into the clinical management of endometriosis and EAOC. This includes the identification of the new biomarkers predictive of the risk of endometriosis and cancer, and it will shape the precision oncology treatment of EAOC.

## 1. Introduction

Endometriosis is a chronic inflammatory disease affecting 5–10% of women of reproductive age worldwide. The most frequent presenting symptoms of endometriosis are pelvic pain, dysmenorrhea, and infertility [1,2]. Endometriosis is a benign condition, characterized by the presence of endometrial glands outside of the uterine cavity. While the pathogenesis of endometriosis is still not completely explained, the most widely recognized hypothesis is that endometriosis develops via retrograde menstruation via fallopian tubes, whereby endometrial cells are spread from the uterus to the peritoneal cavity. However, retrograde menstruation is found in many healthy women who do not ever develop endometriosis. Moreover, endometriosis can develop in women with congenital absence of the uterus (Mayer–Rokitansky–Küster–Hauser syndrome) [3]. Therefore, the hypothesis of retrograde menstruation does not fully explain all the causes of endometriosis. Other hypotheses about the pathogenesis of endometriosis have been proposed, and they include coelomic metaplasia of the peritoneum, hormonal stimulation of ectopic endometrium, oxidative stress DNA damage, and immune dysfunction. While the causes of endometriosis remain unknown, the condition has an estimated heritability of ~50% [4].

Endometriosis can be divided into three anatomical subtypes: deep-infiltrating endometriosis, ovarian endometrioma, and superficial peritoneal endometriosis [5]. The most frequent site of the endometriosis is the ovary, although endometriosis may involve any surface in the body leading to a myriad of symptoms, depending on the location of endometriosis foci. The most common locations of extra-ovarian endometriosis are the recto-sigmoid colon, recto-vaginal septum, and peritoneum [6]. Endometriosis can also lead to lymphangiogenesis, and endometriotic tissue has been found in lymphatic nodes [7,8].

Although it is considered a benign disease, e.g., it lacks the catabolic state typical of malignancy, endometriosis displays some characteristics typical of malignancy, e.g., deep invasion into tissues, spreading, and neoangiogenesis [9] (Table 1). Indeed, a substantial number of observational studies have established a connection between endometriosis and gynecological cancer [10,11]. Ovarian cancer, the most lethal gynecological malignancy, is often associated with endometriosis, especially with the relatively rare histologic subtypes—clear-cell carcinoma of the ovary and endometrioid ovarian adenocarcinoma [12]. This notion was first reported by Sampson in his pioneering report that suggested the existence of cancer arising within endometriosis, i.e., endometriosis-associated ovarian cancer (EAOC) [13]. Sampson, based on microscopic observations, speculated that endometrioid ovarian cancer may develop from endometriotic tissue and described the criteria for the diagnosis of EAOC: (1) evidence of endometriosis close to the tumor, (2) the exclusion of invasion from other sources, and (3) the presence of tissue resembling endometrial stoma surrounding characteristic epithelial glands. In 1953, Scott added a fourth criterion: (4) histological proof of transition from benign changes in endometriosis to malignant changes in cancer [14]. These four criteria are still used today to define endometriosis-associated ovarian cancer. Rarely, endometriosis can be associated with benign tumors such as mucinous cystadenomas and borderline ovarian tumors, including serous, mucinous, and ovarian borderline tumors. Endometrial stromal sarcoma and adenosarcoma can also arise within endometriosis [9].

## 2. Pathogenesis of Ovarian Cancer

Epithelial ovarian cancers (EOCs) comprise five main histologic subtypes: high-grade serous ovarian carcinoma (HGSC) (70%), endometrioid carcinoma (10%), clear-cell carcinoma (10%), mucinous carcinoma (<5%), and low-grade serous carcinoma (LGSC) (<5%) [15]. Prior to the 2000s, it was thought that most or all EOCs arise directly from the surface epithelium of the ovary—a single layer of normally inactive cuboidal cells. The disruption of the cell layer via ovulation and subsequent erroneous repair of the surface epithelium during cell multiplication was considered to be the initiating event in EOC pathogenesis [16].

In 2004, Shih and Kurman proposed a landmark model for a dual pathogenesis of EOC, which accounted for significant differences in the histologic subtypes of EOC [17]. According to these authors, type I EOC comprises EAOC (which can include endometrioid, clear-cell, and seromucinous carcinomas), low-grade serous carcinomas (LGSC), and mucinous carcinomas. These tumors are proposed to develop from implants of extra-ovarian tissue, including endometriosis, that undergoes malignant transformation. Type II EOC are mostly HGSCs, proposed to develop from the fallopian tube epithelium—in particular, from lesions called serous tubal intraepithelial carcinoma (STIC) found in the distal ends of fallopian tubes [17]. Type I carcinomas are frequently low grade (except for clear cell carcinomas) and early stage, with low proliferative rates, while type II more typically present in late stages as high-grade carcinomas.

**Figure 1:**
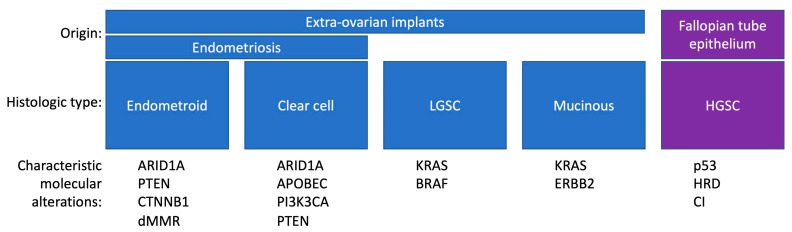
Subtypes of epithelial ovarian cancer arise from distinct precursor lesions. dMMR: Deficient mismatch repair. HRD: Homologous recombination deficiency. CI: Chromosomal instability.

A number of studies have identified the molecular characteristics of type I and type II EOC and helped elucidate the pathogenesis of these tumors (Figure 1). Characteristic molecular features of type I EOC include *ARID1A* loss-of-function mutations, *PIK3CA*-activating mutations, *PTEN* inactivation, microsatellite instability and MMR protein deficiency in endometrioid and clear-cell carcinoma, and KRAS/BRAF/MEK pathway activation in LGSC. Type II EOCs are characterized by high rates of genomic instability and p53 inactivation. In light of the above, the ovarian surface epithelium, fallopian tube epithelium, and extra-ovarian tissue are all thought to play a role in the genesis of ovarian cancer.

Despite their distinct origin, several studies have shown that some susceptibility genes for endometriosis, clear-cell, endometrioid carcinoma and HGSC are shared to some extent [18]. However, the genetic overlap between endometriosis and endometroid and clear-cell carcinoma is much greater than that between endometriosis and HGSC [19]. Mortlock et al. hypothesized that background of endometriosis interacts with context-specific somatic mutations and stromal and hormonal micro-environments to give rise to the distinct histological subtypes of EOC.

Determining the cell(s) of origin for the various subtypes of EOC will have important implications for the diagnosis and prevention of EOC [20,21,22].

## 3. Methods

The study investigators conducted a search in the PubMed database, and the final literature search was completed in December 2023 using the search terms “endometriosis” or “endometriosis associated ovarian cancer” in combination with “genetic”, “genetic alterations”, and “tumor microenvironment”.

### 3.1. Epidemiologic Studies

The lifetime risk of developing ovarian cancer is low for patients with endometriosis (~1.9%), but it is relatively increased compared to the risk in the general female population (lifetime risk of approximately 1.4%) [23]. Epidemiologic studies have established a link between a history of endometriosis and the occurrence of EOC. In a register study of 20,686 Swedish patients hospitalized with endometriosis, the standardized incidence ratio (SIR, the ratio of observed to expected cases multiplied by 100) for developing ovarian cancer during a mean follow up of 11.4 years was 1.9 (95% CI: 1.3–2.8) [24]. The risk of EOC was higher in patients with a longer history of endometriosis. In another Swedish register study including 64,492 women with endometriosis, the SIR for ovarian cancer was 1.43 (95% CI: 1.19–1.71), again with a higher incidence in women with an earlier diagnosis and a longer history of endometriosis [25]. In a retrospective cohort study of 12,193 women, Brinton et al. found the SIR for ovarian cancer was increased to 4.2 in women with a combination of endometriosis and primary infertility [26]. Additionally, a meta-analysis that had included 24 observational studies evaluating the association between endometriosis and ovarian cancer calculated a summary relative risk of ovarian cancer of 1.93 (95% CI: 1.68–2.22) among women with endometriosis compared with those without endometriosis [27].

Large epidemiologic studies have found that the clear-cell and endometrioid subtypes of ovarian cancer are most strongly associated with a history of endometriosis, followed by LGSC. A 20-year population-based Danish cohort study of more than 99,000 patients found a relative risk of clear-cell ovarian cancer of 3.37 (95% CI: 1.24–9.14) and endometrioid ovarian cancer of 2.53 (95% CI: 1.19–5.38) among patients diagnosed with endometriosis five or more years prior, with no association between endometriosis and other ovarian cancer subtypes, including LGSC [28]. Similarly, a case–control study of ovarian cancer patients in the United States in Washington state demonstrated an association of endometriosis with clear-cell and endometrioid ovarian carcinomas with an odds ratio (OR) of 2.8 (95% CI 1.7–4.7) and no association with other ovarian cancer histologic subtypes [29]. In contrast, in 2012, the Ovarian Cancer Association Consortium (OCAC) summarized pooled data from 13 case–control studies, with over 23,000 women who self-reported endometriosis, and found an increased risk of not only clear-cell and endometrioid ovarian cancer but also of LGSC [30].

In order to correct for inherent bias in meta-analyses that may lead to the overestimation of risks, a 2023 study by Wang et al. utilized a two-sample Mendelian randomization method and nevertheless still found a strong correlation between endometriosis and ovarian cancer with an OR of 1.23 (95% CI: 1.11–1.36) [31]. Further histologic subtype analyses revealed a strong association of endometriosis with the risk of endometrioid carcinoma (OR 1.49, 95% CI 1.24–1.81), clear-cell carcinoma (OR 2.56, 95% CI 1.75–3.73), and low-malignant-potential ovarian tumors (OR 1.28, 95% CI 1.08–1.53).

Interestingly, a Finnish population-based study in 2018 by Saavalainen et al. assessing the risk of EAOC by endometriosis subtype found that ovarian endometriosis specifically was associated with an increased risk of endometrioid (SIR 4.72, 95% CI: 2.7–7.56) and clear-cell ovarian cancer (SIR 10.1, 95% CI: 5.50–16.9), while peritoneal and deep-infiltrating endometriosis were not associated with an overall increased risk of ovarian cancer [32,33]. However, a recent study of deep-infiltrating endometriosis found frequent somatic mutations in the cancer driver genes *KRAS*, *TP53*, and *ATRX* more than in other, non-driver cancer genes, bringing up the question of the role of *KRAS*, in particular in the development of endometriosis (without cancer), and the need to carefully follow up with patients with deep endometriosis [34].

Within ovarian endometriosis, the endometrioma type may also affect the risk for the development of ovarian cancer. Nezhat et al. have described two types of endometriomas: type I are small (3 cm or smaller), densely fibrotic cysts that form from ovarian surface endometriosis that bleeds into the ovarian cortex, while type II endometriomas are large (greater than 3 cm) cysts that form from the invasion of functional cysts by endometriosis [35,36]. In premenopausal women, large type II endometriomas occur only in the ovaries and are associated with an increased risk of cancer [35]. Gilbert et al. investigated the clonal origin of five non-cancerous endometriotic cysts by a recombinant DNA X-chromosome inactivation technique and found that three cysts showed a monoclonal inactivation pattern, i.e., the cell populations of those cysts were each derived from one cell, suggesting that endometriotic cysts can, in at least some cases, be regarded as clonal processes [37]. Other predictive factors of the risk of ovarian cancer among endometriosis patients include menopausal status and hyperestrogenism, both endogenous and exogenous [38]. In a study by Zanetta et al., obesity as well as therapy with unopposed estrogens after hysterectomy were shown to be significant risk factors for the development of EAOC [39].

Genome-wide association studies (GWASs) have suggested a strong shared germline genetic susceptibility to both endometriosis and EOAC (Table 2). Mortlock et al. assessed the causal relationship between genetic endometriosis liability and EOC histologic subtypes using GWAS meta-analysis data. Their extensive analyses included genetic correlation, Mendelian randomization, bivariate GWAS, colocalization, and functional genomic analyses. Using the comprehensive analysis, they found a strong and significant genetic correlation (r_g_) between endometriosis and clear-cell carcinoma (r_g_ = 0.71), with positive but weaker associations with endometriosis and endometrioid carcinoma (r_g_ = 0.48) and endometriosis and HGSC (r_g_ = 0.19) [40]. They further identified 19 genetic susceptibility loci associated with both endometriosis and EOC. Other genome-wide association studies have identified significant genetic correlations between endometriosis and endometrioid and clear-cell carcinoma in particular, with a weaker genetic overlap with LGSC and yet weaker correlation with HGSC [40,41].

### 3.2. Histology and Pathogenesis of Endometriosis-Associated Ovarian Cancer

**Figure 2:**
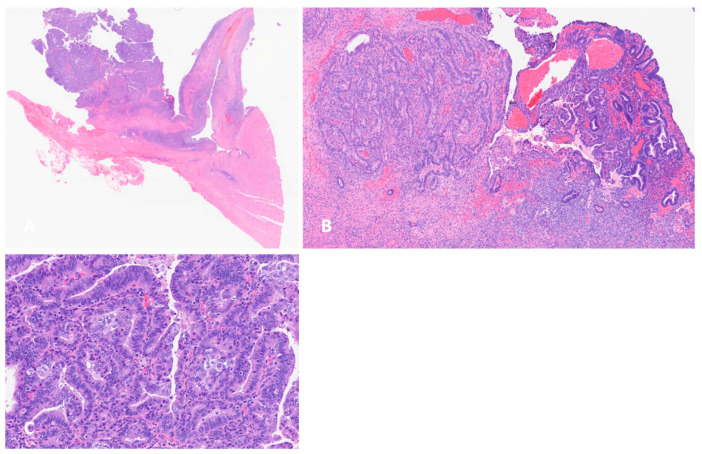
A 4.8 cm ovarian mass showing transition from simple cyst lining to papillary and glandular outgrowth (H&E, 10× mag) (**A**). Areas of the cyst lining showing endometriosis (left side) transforming into atypical endometrial (endometriosis with atypical hyperplasia) (H&E, 50× mag) (**B**). Endometrioid carcinoma, FIGO grade 1, arising adjacent to the atypical endometriosis (H&E, 200× mag) (**C**).

Histologically, EAOC can be found in coexistence with benign typical endometriosis and/or atypical endometriosis or may be found without histologically confirmed endometriosis [42,43,44] (Figure 2). Atypical endometriosis is defined by several histologic criteria, including tall crowded epithelial cells (architectural atypic) exhibiting an increased nuclear/cytoplasmic ratio (cytologic atypia), mild hyperchromia, and mild to moderate pleiomorphism [43]. Architectural atypia, also called hyperplasia, has the strongest association with EAOC. Atypical endometriosis is identified in up to 4% of all endometriosis lesions and in 12–35% of ovarian endometriosis. Histologic EAOC specimens have been identified to demonstrate a continuum of transition from benign to atypical endometriosis to EAOC [42,43,45,46,47]. Due to these histologic findings and subsequent molecular data suggesting a clonal relationship between atypical endometriosis and EAOC (discussed in more detail below), atypical endometriosis has been suggested as the putative precursor of EAOC, but nevertheless is not identified in all cases of EAOC [48]. Wepy et al. recently reviewed and analyzed over 4500 cases of endometriosis and identified 36 cases of atypical endometriosis [49]. Of the 36 cases, 9 had associated endometrioid, sero-mucinous, and clear-cell tumors (borderline and invasive). In that study, gland crowding and Napsin A positivity differentiated atypical from typical endometriosis and were recognized as risk factors for EAOC. Sequencing showed pathogenic variants in five of six cases analyzed, involving mutations in *ATM*, *BRCA2*, *KRAS*, *AKT*, *CTNNB1*, *PTEN*, and *ARID1A*. *TP53* was wild-type in all cases, and mismatch repair was intact [49]. Interestingly, proteomic studies have suggested that ovarian endometrioid carcinoma arises from the secretory cells of endometriosis, while clear-cell carcinomas arise from ciliated cells [50].

### 3.3. ARID1A Mutations and Other Genetic Alterations

The link between endometriosis and EAOC has been described at the molecular level, including the presence of common mutations in the cancer-associated genes: AT-rich interaction domain 1A (*ARID1A*), phosphatidylinositol-4,5-bisphosphate 3-kinase catalytic subunit alpha (*PIK3CA*), phosphatase and tensin homolog (*PTEN*), *PPP2R1A*, *ERBB2*, *MLH1*, and Kirsten rat sarcoma (*KRAS*) [19,51,52]. *PIK3CA* and *PTEN* are both related to the PI3K/AKT/mTOR (PI3K) pathway, which is involved in various cellular functions associated with carcinogenesis. PIK3CA is considered to be oncogene, while PTEN functions as a negative regulator of the PI3K pathway and acts as a tumor-suppressor gene. *PIK3CA* and *KRAS* mutations have been demonstrated to be clonally expanded in endometriosis [53]. *KRAS* codon 12-activating mutations have been shown to be associated with the proliferation, local invasion, and metastatic spread of endometriosis and large disease burden resulting in advanced-stage disease with increased surgical complexity [54]. The RAS/RAF/MEK (MAPK, mitogen-activated protein kinase) signaling pathway may therefore be a relevant target to reduce the invasiveness, spread, or burden of endometriosis.

The *ARID1A* gene encodes ARID1A/BAF250A, a key subunit of the SWI/SNF (SWItch/Sucrose Non-Fermentable) chromatin remodeling complex. In addition to *ARID1A* mutation, mutations of *ARID1B* and *SMARCA4* affect the SWI/SNF complex. The SWI/SNF complex plays an important role in chromatin remodeling and is associated with numerous biological functions, such as differentiation and proliferation [55]. The SWI/SNF complex binds to DNA regions via ARID1A or ARID1B, which are mutually exclusive, nonselective DNA-binding accessory subunits of the complex [56,57]. Depending on the cell type and time-point in development, these complexes can both repress and activate gene expression [58].

*ARID1A* pathogenic mutations are, in general, loss-of-function mutations (nonsense, frameshift, and large deletions) that lead to a loss of BAF250. Consequently, the *ARID1A* gene is considered to be a tumor-suppressor gene. ARID1A protein immunohistochemistry has been shown to be an excellent surrogate marker for *ARID1A* mutation [51,59]. This is particularly helpful as *ARID1A* is a large gene consisting of 20 exons and its sequencing may be challenging. Of note, endometriosis is the only known benign disease with the loss of *ARID1A* expression. Most studies suggest that *ARID1A* mutation alone does not seems sufficient for the malignant transformation of endometriosis [52,60,61]. Additional mechanisms are needed to co-work with *ARID1A* loss of function in endometriosis (benign or atypical) to initiate the process of carcinogens in clear-cell ovarian cancer. These include the activation of the PIK3CA pathway via gain-of-function mutations of the PIK3CA gene [62,63]. Chandler et al. suggested that ARID1A-deficient tumors may be “addicted” to PI3K/AKT oncogenic signaling [64].

On the other hand, *ARID1A* mutations have been observed in benign endometriosis and in atypical endometriosis contiguous with clear-cell ovarian cancer, but not in endometriotic lesions distant from EAOC. Therefore, some groups favor the concept that *ARID1A* mutation may be an early event in the transformation from endometriosis to clear-cell ovarian cancer [51,62].

While *ARID1A* mutations are present in about 45% of EAOCs, there are no known *ARID1A* mutations that distinguish clear-cell carcinoma versus endometrioid ovarian carcinomas [3]. However, there is evidence that microsatellite instability (MSI) is present in a higher proportion (~28%) of endometrioid ovarian carcinomas, while clear-cell carcinomas more frequently show APOBEC signatures (~26%) [3]. Furthermore, in clear-cell ovarian cancer, *ARID1A* mutations frequently co-occur with mutations that lead to the activation of the PI3K/AKT signaling pathway, such as *PIK3CA*-activating mutations or *PTEN*-inactivating mutations [63]. These changes have also been observed in benign and atypical endometriosis adjacent to clear-cell ovarian carcinoma and suggest a cooperative role of *ARID1A* inactivation and PI3K/AKT activation in the malignant transformation of the endometriotic precursor lesion [65]. In cases of endometriosis associated with LGSC, about one-third of LGSCs show *KRAS* mutations, in comparison with LGSCs without endometriosis where the rate of *KRAS* mutations is only 3% [66].

SWI/SNF function is also required for resistance to oxidative stress, which likely plays a role in the pathogenesis of EAOC [64]. A hypothetical two-hit model of malignant transformation of endometriosis suggests that reactive oxygen species produced by free heme and iron in the trapped blood in endometriomas may lead to increased oxidative stress and DNA damage of the epithelial cells of endometriomas. Erroneous DNA replication may lead to the accumulation of inactivating genetic mutations in cancer genes such as *ARID1A* and activating mutations in *PI3K*, ultimately leading to carcinogenesis [3,67]. In this two-hit model, *ARID1A* mutation would represent the initial, first-hit genetic event, while mutations in *PI3K* represent the second genetic hit, initiating invasive carcinogenesis.

In endometriosis and in clear-cell ovarian carcinoma, *ARID1A* mutations are also frequently accompanied by the loss of *PTEN* (phosphatase and tension homolog) [68]. *PTEN* is located on chromosome arm 10q23 and is considered a tumor-suppressor gene. *PTEN* regulates the PI3K/AKT pathway, and studies have shown that the decrease in *PTEN* function activates the PI3K/AKT pathway via the accumulation of PIP3 [69]. In ovaries, the loss of PTEN in tubal epithelial cells is sufficient to induce tubal cancer, with the subsequent involvement of the ovaries in tubo-ovarian cancer. Additionally, homozygous *PTEN* deletion produces borderline serous tumors of the fallopian tube epithelium (FTE) and endometriosis-associated carcinoma [70].

*PPP2R1A* gene (protein phosphatase 2 scaffold subunit alpha) mutations have been described in 4% of clear-cell ovarian carcinomas and 12% of endometroid ovarian carcinomas, but they are not documented in HGSC or in LGSC [71,72]. Interestingly, in two clinical studies, among 28 patients with clear-cell carcinomas, those with somatic *PPP2R1A* had a remarkable response to treatment with immune checkpoint inhibitors [73].

Human epidermal growth factor receptor 2, *ERBB2* (*HER2*), has been extensively studied in breast cancer, where it has both prognostic and therapeutic importance. Its role in ovarian cancer is less clear [74]. *HER2* is more frequently expressed (43%) in ovarian clear-cell carcinoma than in other histologic subtypes [75]. While ovarian carcinomas, including the clear-cell type, have shown poor responses to herceptin, the recent data from the Phase 2 Destiny clinical trial with a novel antibody drug conjugate, trastuzumab deruxtecan, have revealed a remarkable response rate of 45% in a cohort of 40 ovarian carcinomas, including complete responses, though histologic subgroup outcomes have not been reported [76]. The responses were documented in tumors with high expression levels of HER2 (2+, 3+), regardless of amplification.

MMR deficiency (MMRd) has been reported in 10–15% of epithelial ovarian carcinomas, which included 35% with clear cell, 34% with endometrioid, 26% with mucinous histology, and 0 with serous histology [77]. *MSH6* mutation was the most frequent (55%). The prevalence of MMR protein deficiency was found to be 15% in non-serous ovarian carcinomas [78].

Defective MMR leads to the accumulation of mutations in the genome and microsatellite instability in tumors, suggesting potential benefits to the therapeutic use of immune-checkpoint inhibitors [79].

In women with hereditary Lynch Syndrome, the risk of ovarian cancer is 6–12%, with clear-cell carcinoma and endometrioid carcinoma being typical subtypes. Among these patients, the most frequent mutations are *MLH1* (38%) and *MSH2* (47%) [80].

### 3.4. Microenvironment of Endometriosis and EAOC

The peritoneal microenvironment of endometriosis consists of epithelial, stromal, and immune cells [81]. This environment is hypoxic with high levels of estrogen and iron, necessitating adaptation for cell survival [82]. Hypoxia induces HIF1A expression, the downregulation of DUSP2, and ERK activation, which ultimately activates angiogenesis and proliferation [83]. VEGF and leptin have also been implicated in the establishment of the vascular network in endometriosis [84,85,86]. In addition to hypoxia-induced angiogenesis, endometriotic tissue undergoes inflammation and the increased expression of tissue factor (TF) during menstrual cycles. TF is an important factor in the extrinsic coagulation pathway and leads to a hypercoagulable state; this molecular pathway has been suggested to explain the observation that patients with clear-cell carcinoma often present with deep venous thrombosis or pulmonary embolism [87].

The interaction of the immune system and EAOC is still being elucidated. In endometrioid ovarian carcinoma, immune cell infiltrates are correlated with the tumor molecular subtype, with higher immune infiltration in more highly mutated tumors, but immune infiltration is not independently correlated with prognosis [88]. Among four different molecular subtypes (POLEmut, MMRd, P53+, and NSMP), three distinct TIL patterns (hot, cold, and TILb-) were distributed differentially across stromal and epithelial compartments. POLE-mutated and MMR-deficient subgroups were enriched for TIL hot tumors; P53 abnormal tumors had low densities of CD8+ T cells in both stromal and epithelial compartments, while CD3+/CD8− T lymphocytes were the only cells in the stoma of NSMP tumors. The prognostic significance of immune clustering was described only for the NSMP subgroup, where the group of tumor infiltrates lacking B cells (TILB-) had poor outcomes [88].

Clear-cell carcinoma has been shown to produce an immunosuppressive state via HNF-1β activation [89]. Substantial PD-L2 expression has been demonstrated to be localized to malignant cells within the clear-cell carcinoma microenvironment, with limited PD-L1 expression [90,91]. *ARID1A* mutant clear-cell ovarian carcinoma and associated endometriosis have been shown to be highly enriched in mast cells, CD20+ B cells, and CD138+ plasma cells, divergent from *ARID1A* wild-type clear-cell ovarian tumors, which lack these infiltrates [90]. This may suggest that different subtypes of ovarian clear-cell carcinoma develop along different pathways that depend on the interaction of *ARID1*mut and *ARID1*wt with the TME.

Of note, clinical studies of immune checkpoint blockades have typically returned impressive results in advanced endometroid endometrial cancer but disappointing results in ovarian HGSC. The resistance to immune checkpoint blockades is caused by the low density of tumor-infiltrating lymphocytes, low tumor mutational burdens, and low microsatellite instability [92]. In particular, EAOCs are genetically stable tumors. Yet, emerging evidence has suggested that about 20% of clear-cell ovarian cancers may be more responsive to immune checkpoint blockades [93,94,95]. Further elucidation of the interaction between the immune system and EAOC may provide important insights as to selecting the patients with clear-cell carcinoma who would most likely respond to immune checkpoint inhibitors.

Endometriosis is a complex disease with a complex interplay between genetic factors and multiple factors within the tissue microenvironment that are influenced by epigenetic, post-translational, and other changes. Modifications such as DNA methylation, histone deacetylation and hypermethylation, and non-coding microRNAs have been described to contribute to differential gene expression within endometriotic foci via their repressive or activating influence on the genome. These factors are under investigation and may represent useful targets for the future treatment of endometriosis and EAOC. At this time, no epigenetic alteration of genetic loci has been identified as a predictive marker for the malignant transformation of endometriosis.

EAOC is characterized by chronic inflammation and exhibits high levels of pro-inflammatory cytokines, including IL-6. Il-6 is thought to promote carcinogenesis via interacting with the PI3K/AKT/mTOR pathway [64]. A group identified IL-6 as a direct target of the *ARID1A* tumor suppressor and suggested that in the absence of the negative regulation of *ARID-1A*, the coexisting amplification of PIK3CA promotes IL6 over-expression, thus maintaining the JAK/STAT signaling loop, which in turn positively interconnects with the mTOR pathway. Therefore, the synergistic action of ARID1A loss and PI3K/AKT/mTOR pathway upregulation promote the malignant progression of endometriosis [96].

### 3.5. The Role of Micro RNAs

Micro RNAs (which are non-coding RNAs) regulate mRNA expression or the degradation of target genes at the post-translational level. About one thousand miRNAs have been cloned in the human genome, and they usually act in concert to modify the expression of the target mRNA. They have been recognized to have a role in the malignant transformation of endometriosis [97].

In endometriosis, high miR-15b/16, miR-143, and miR-145 and low miR-20a, miR-221, and miR-222 expression are consistent with repressed cell proliferation and enhanced cell survival [98]. In cancer, miRNAs and their targets may have both ontogenetic and tumor-suppressive functions, and therefore, miRNA-mediated regulation is highly complex in cancer. miRNA-125a and miRNA-125b are tumor-suppressor miRNAs that repress the ERBB2 (HER2) and ERBB3 (HER3) oncogenes [99].

The loss of miR-125a/b activity is associated with elevated ERBB2 levels seen in ovarian endometrioid adenocarcinomas arising from endometriosis [100].

*PTEN* is regulated by various microRNAs, including miRNA-200a, miR-205, and miR-552 and miR-21. The overexpression of mIR-221 downregulates *PTEN* (and thereby promotes the carcinogenesis process of EAOC [101].

Ohlsson et al. reported a significant decrease in the expression of the miR-200 family, leading to the epithelial–mesenchymal transition characteristic of endometriosis [102].

Numerous other miRNAs play important roles in endometriosis and EAOC, including miRNA, e.g., 191, miRNA 20a, and miRNA 143. One important aspect of further research on the role of miRNA is that they have potential to serve as predictive markers for endometriosis and EAOC.

**Table 2:** Epidemiologic genetic risk of endometriosis-associated ovarian cancer *.

Study	Type	
Lee et al., 2016 [103]	GWAS	SNP Rs7515106 (chr 1p36)
Sapkota et al., 2017 [104]	GWAS	SNPs at *FN1*, *CCDC170*, *ESR1*, *SYNE1*, *FSHB* (*sex steroid hormone pathway*)
Phelan et al., 2017 [105]	GWAS	SNP at 5q12.3
Lu et al., 2015 [41]	SNP	
Mortlock et al., 2022 [40]	GWAS	28 SNPs associated with both endometriosis and ovarian cancer (chr 1, 2, 4, 5–10, 1217, 18)

* The list is not exhaustive.

## 4. Conclusions and Clinical Implications

In this review, we summarized the current evidence regarding the epidemiologic genetic links between endometriosis and ovarian cancer and the molecular genetic evidence of progression from endometriosis to ovarian cancer.

EAOC is an important health problem. Five million patients with endometriosis in the United States are potentially at risk of ovarian cancer. EAOC usually presents in early stages, and endometrioid ovarian cancer is typically a low-grade malignancy; however, clear-cell carcinoma of the ovary is a high-grade cancer that is often chemotherapy-resistant. Patients with EAOC are usually 10 years younger than patients with the much more frequent HGSC [106]. The prognosis of EAOC does not differ from other non-endometriosis-related ovarian cancers, when controlled for stage and age [107]. All these elements are important to consider in the clinic when assessing patients with endometriosis. Clinicians should be aware of the increased risk of specific subtypes of ovarian carcinoma in patients with endometriosis and discuss the increase in cancer risk in light of the risk factors for malignant transformation—specifically, the duration and type of endometriosis lesions and hormonal status [9]. The possibility of malignant transformation should be included in diagnostic and treatment considerations for patients with endometriosis. The frequent clinical manifestations of increased risk are long-standing endometriosis, history of infertility, abdominal mass and abdominal pain, and the rapid growth of endometrioma. Imaging, in particular magnetic resonance of the pelvis, may suggest malignant transformation by revealing mural modularity within ovarian endometrioma.

It is important to give patients with endometriosis a balanced view of the risk of ovarian cancer, and it is critical to inform patients about the risk using evidence-based principles. The population risk of EOC is 1 in 76 female patients, and that risk is slightly increased among patients with endometriosis to 1 in 55 female patients. While this is reassuring, clinicians should be aware of the risk factors in patients with endometriosis that may suggest a higher-than-average risk of EOC and discuss risk reduction.

With advancements in diagnostics, surgical techniques, as well as molecular genetic analysis, the more precise definition of atypical endometriosis, in the future, it may be increasingly possible to quantify the risks of ovarian cancer for women with endometriosis. This capability would mark significant progress in resolving management dilemmas for both clinicians and patients with endometriosis. The identification of the risk factors for endometriosis and EAOC, which are possibly modifiable, will have significant impacts on public health. The prospect of the noninvasive diagnosis of endometriosis is here, and several potential biomarkers are being investigated, including cytokines and inflammatory mediators, as well as metabolomics and proteomics and transcriptome analyses. However, none of them have been proven to be a sensitive and reliable diagnostic biomarker. The frequency of endometriosis, the occurrence of EAOC in patients at the prime of their lives, and the chemotherapy resistance of clear-cell ovarian carcinoma all emphasize the urgent need for the continued investigation of the pathogenesis and treatment of this important clinical entity.

## Figures and Tables

**Table 1:** Similarities * between endometriosis and ovarian cancer.

	Endometriosis	Ovarian Cancer
Early menarche	yes	yes
Infertility	yes	yes
Estrogen exposure	yes	yes
Family history	yes	yes
Nulliparity	yes	yes
BTL (bilateral tubal ligation)	protective	protective
Hysterectomy	protective	protective
Progesterone exposure	protective	protective
Symptoms	yes	yes
Elevated CA125	yes	yes
Invasion/infiltration of tissues	yes	yes
Spreading	yes	yes
Neoangiogenesis	yes	yes
Genetic alterations	yes	yes

* The main difference between endometriosis and cancer is that cancer is a highly proliferative and catabolic state, while endometriosis lacks these characteristics. Clinically, pain in endometriosis is cyclic, while in ovarian cancer, it is dull and steady.
